# *O*-GlcNAcylation and its role in the immune system

**DOI:** 10.1186/s12929-020-00648-9

**Published:** 2020-04-29

**Authors:** Yi-Hsuan Chang, Chia-Lin Weng, Kuo-I Lin

**Affiliations:** 1 Genomics Research Center, Academia Sinica, 128 Academia Road, Sec. 2, Nankang Dist., Taipei, 115 Taiwan; 2 Graduate Institute of Immunology, College of Medicine, National Taiwan University, Taipei, 110 Taiwan

**Keywords:** *O*-GlcNAcylation, OGT, OGA, Immune cells

## Abstract

*O*-linked-N-acetylglucosaminylation (*O*-GlcNAcylation) is a type of glycosylation that occurs when a monosaccharide, *O*-GlcNAc, is added onto serine or threonine residues of nuclear or cytoplasmic proteins by *O*-GlcNAc transferase (OGT) and which can be reversibly removed by *O*-GlcNAcase (OGA). *O*-GlcNAcylation couples the processes of nutrient sensing, metabolism, signal transduction and transcription, and plays important roles in development, normal physiology and physiopathology. Cumulative studies have indicated that *O*-GlcNAcylation affects the functions of protein substrates in a number of ways, including protein cellular localization, protein stability and protein/protein interaction. Particularly, *O*-GlcNAcylation has been shown to have intricate crosstalk with phosphorylation as they both modify serine or threonine residues. Aberrant *O*-GlcNAcylation on various protein substrates has been implicated in many diseases, including neurodegenerative diseases, diabetes and cancers. However, the role of protein *O*-GlcNAcylation in immune cell lineages has been less explored. This review summarizes the current understanding of the fundamental biochemistry of *O*-GlcNAcylation, and discusses the molecular mechanisms by which *O*-GlcNAcylation regulates the development, maturation and functions of immune cells. In brief, *O*-GlcNAcylation promotes the development, proliferation, and activation of T and B cells. *O*-GlcNAcylation regulates inflammatory and antiviral responses of macrophages. *O*-GlcNAcylation promotes the function of activated neutrophils, but inhibits the activity of nature killer cells.

## Background

### The discovery and synthesis of *O*-GlcNAcylation

**Fig. 1:**
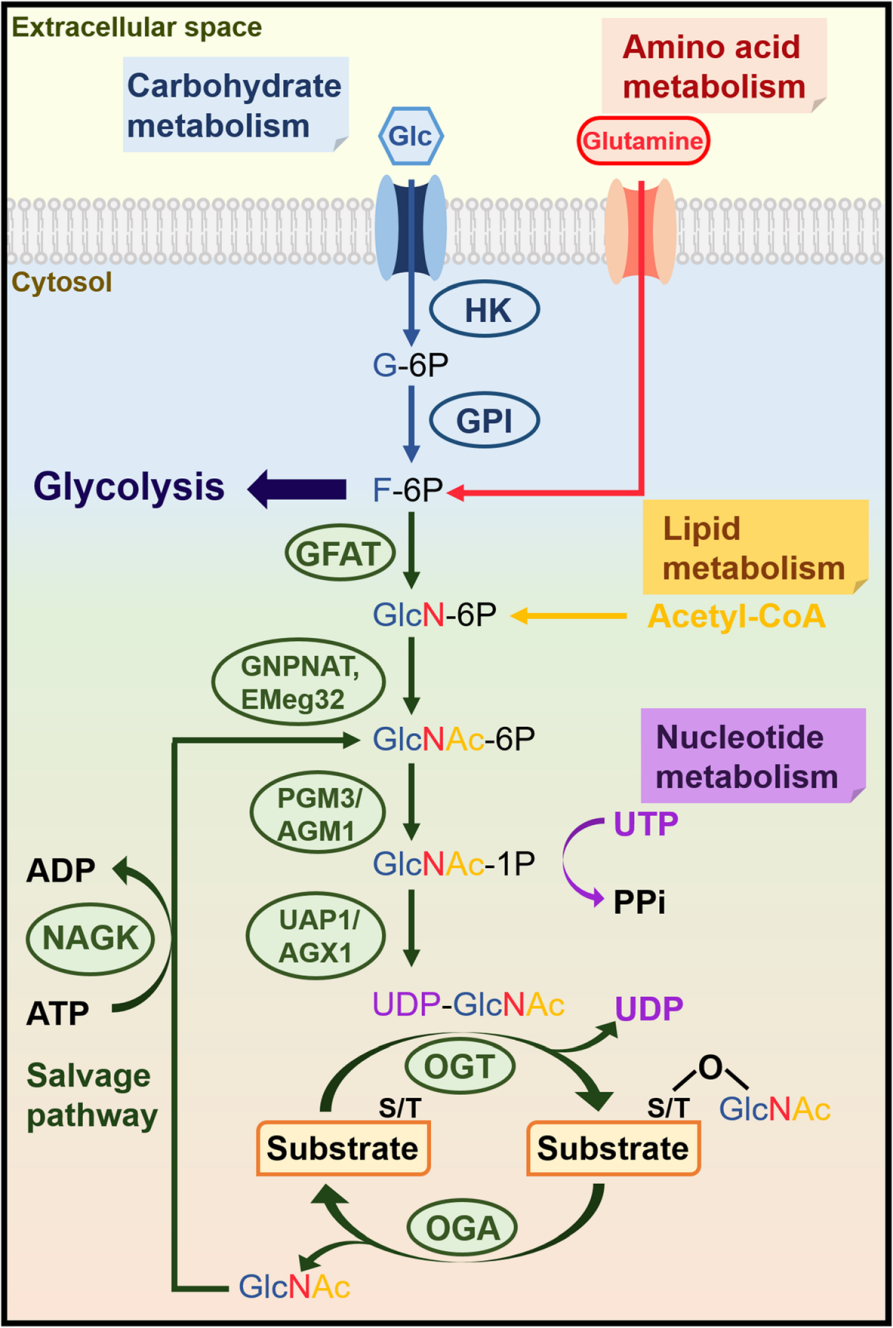
Overview of the Hexosamine Biosynthetic Pathway (HBP) and *O*-GlcNAcylation. The HBP integrates four metabolism pathways, including carbohydrate (glucose), amino acid (glutamine), lipid (Acetyl-CoA) and nucleotide (UTP). Glucose becomes F-6P through the initial two steps shared by the HBP and glycolysis pathway. Only 2–3% F-6P enters the HBP, and in combination with glutamine, Acetyl-CoA and UTP generates UDP-GlcNAc. OGT catalyzes the transfer of GlcNAc moiety onto the serine (S) or threonine (T) site on the protein substrates, while OGA is able to remove the GlcNAc. Free GlcNAc can return to the HBP through the salvage pathway.

Protein *O*-GlcNAcylation was first discovered by Hart and Torres using bovine milk galactosyltransferase to conjugate tritiated UDP-galactose on *N*-acetylglucosamine (GlcNAc) residues on the surfaces of murine lymphocytes nearly 35 years ago [1]. This is a type of post-translational modification by glycosylation that links a single GlcNAc molecule to the serine/threonine (S/T) site on proteins by a *O*-linked β-glycosidic bond [2]. The addition and removal of monosaccharides is regulated by *O*-GlcNAc transferase (OGT) and *O*-GlcNAcase (OGA), respectively (Fig. 1). OGT, originally detected in rabbit reticulocytes and purified from rat liver cytosol, is responsible for transferring GlcNAc derived from uridine diphosphate *N*-acetylglucosamine (UDP-GlcNAc) to protein S/T residues upon the release of UDP [3]. OGA was first purified from rat spleen cytosol and found to be able to mediate the removal of GlcNAc from proteins [4]. Unlike other types of protein glycosylations, which are mainly produced by the secretory pathways, the subcellular distribution of *O*-GlcNAcylation is ubiquitous in the nucleus and cytoplasm as only about 7% of *O*-GlcNAc moieties are detected on the cell surface [1, 5]. *O*-GlcNAcylation was shown to be highly enriched on the proteins in nuclear pore complexes and nuclear envelopes [2, 6–8], as well as on proteins interacting with chromatin [9]. Moreover, cytoskeletal proteins [10], and intrinsic membrane proteins of the Golgi apparatus and endoplasmic reticulum (ER) were also verified to possess *O*-GlcNAc moiety [11, 12].

UDP-GlcNAc, which serves as the donor substrate for *O*-GlcNAcylation is generated from the hexosamine biosynthetic pathway (HBP) (Fig. 1). The HBP uses glucose in combination with glutamine, acetyl-coenzyme A (Acetyl-CoA) and uridine-5′-triphosphate (UTP), which are derived from amino acids, lipids and nucleotide metabolic pathways, respectively, to generate UDP-GlcNAc [13, 14]. Only approximately 2–3% of cell glucose uptake enters the HBP [15]. The first two steps in the HBP, phosphorylation of glucose to become glucose-6-phosphate (G-6P) by hexokinase (HK), and phosphoglucose isomerase (GPI)-mediated transformation into fructose-6-phosphate (F-6P), are shared with glycolysis. Instead of entering glycolysis, a small amount of F-6P is committed to be transaminated with glutamine by glutamine:fructose-6-phosphate amidotransferase (GFAT) to become glucosamine-6-phosphate (GlcN-6P), which is the rate-limiting step of the HBP [15, 16]. In the presence of Acetyl-CoA, GlcN-6P is then acetylated by glucosamine-phosphate *N*-acetyltransferase (GNPNAT, EMeg32) to produce *N*-acetylglucosamine-6-phosphate (GlcNAc-6P) [17], which further undergoes isomerization by GlcNAc phosphomutase (PGM3/AGM1) to produce *N*-acetylglucosamine-1-phosphate (GlcNAc-1P) [18]. Finally, GlcNAc-1P and UTP are converted into UDP-GlcNAc and pyrophosphate (PPi) via UDP-GlcNAc pyrophosphorylase (UAP1/AGX1) [19]. Furthermore, free GlcNAc removed from proteins by OGA can re-enter the HBP via the salvage pathway, in which GlcNAc is converted into GlcNAc-6P by *N*-acetylglucosamine kinase (GlcNAc kinase, NAGK) [20, 21]. Overall, the intracellular level of UDP-GlcNAc is influenced by the metabolic pathways and as a result, HBP and *O*-GlcNAcylation are considered as the intracellular nutrient sensors [13, 14, 22]. For instance, glucose deprivation results in reduced cell growth and decreased levels of UDP-GlcNAc in growth factor treated cells [23]. Activated T cells cultured in medium lacking glucose or glutamine show relatively reduced levels of intracellular UDP-GlcNAc [24]. Similarly, glucose deprivation leads to decreased levels of intracellular UDP-GlcNAc and reduced levels of protein *O*-GlcNAcylation, which in turn results in upregulation of OGT mRNA transcription in the HepG2 human liver cancer cell line [25]. Saturated fatty acids, such as palmitate and stearate, are sources of Acetyl-CoA and trigger increased expression of GFAT in human myotubes, which in turn induces enhanced intracellular concentrations of UDP-GlcNAc [26]. Moreover, the addition of GlcNAc into medium caused accumulation of UDP-GlcNAc, and changes in intracellular UDP-GlcNAc levels, which influenced cell growth [27].

Although UDP-GlcNAc has long been viewed as the sensor for nutrients, a study on 3T3-L1 adipocytes showed that fluctuations in UDP-GlcNAc concentration may not fully reflect changes in extracellular glucose amounts because treatment with 1 to 25 mM glucose upregulated UDP-GlcNAc at similar levels (all about 1.5- to 2-fold more than that in untreated cells) [28]. This may be explained by the fact that UDP-GlcNAc is usually concentrated in the ER and Golgi for initiation and branching endomembrane glycosylation, respectively [29, 30]. When the capacity of UDP-GlcNAc in these two organelles reaches saturation, free UDP-GlcNAc is released into the cytosol, nucleus and mitochondria. Conversely, if the cellular level of UDP-GlcNAc remains low, the majority of UDP-GlcNAc is conserved in the ER and Golgi apparatus, leading to lower UDP-GlcNAc levels in the cytoplasm and nucleus [22]. In addition, the rate limiting enzyme GFAT is negatively modulated by downstream products, GlcN-6P and UDP-GlcNAc, which prevent the generation of excessive UDP-GlcNAc [15, 16]. In summary, the levels of UDP-GlcNAc in different organelles reflect the metabolic situation of the cells, which is significant for maintaining cell viability and homeostasis.

### Enzymes involved in *O*-GlcNAc cycling: OGT and OGA

The *OGT* gene resides on the X chromosome and is indispensable for embryonic stem cell survival and development [31–33]. OGT is ubiquitously expressed [31, 32, 34]. The open reading frames of the *OGT* gene are highly conserved in several species including rats, mice, rabbits, *C. elegans* and humans [34, 35]. The OGT transcript encodes a protein with two major regions. The N-terminal domain is composed of a series of superhelical tetratricopeptide repeats (TPRs) whose repetitive number depends on the species and type of alternatively spliced isoforms. The C-terminal region comprises multiple catalytic domains [31, 34–36]. Human OGT encodes three isoforms, which results from the alternative splicing of transcripts in the N-terminal region. Full-length human OGT, also called nucleocytoplasmic OGT (ncOGT), contains 13 TPRs and has the molecular weight of 116 kDa. While mitochondrial OGT (mOGT) has the molecular weight of about 103 kDa and only 9 TPRs along with an alternatively spliced N-terminal mitochondria targeting sequence. The shortest form of OGT (sOGT), which has a molecular weight of approximately 78 kDa, possesses only 2 TPRs [31]. Like the distribution of protein *O*-GlcNAcylation, ncOGT and sOGT are located in the nucleus and cytoplasm; however, mOGT tends to accumulate in the mitochondrial inner membrane [37].

Full-length OGT exists as a trimer [38]. TPRs help OGT multimerization because the deletion of TPRs 1–6 abrogates the subunit assembly [39]. Structural analysis of the human OGT protein revealed that the TPR domains usually form a dimer of superhelices. Mutants on the dimerization interface of TPR superhelices prevent the formation of TPR dimers in solution and cause moderate reduction of the enzymatic activity of OGT toward nucleoporin p62 [40]. Wild-type OGT has three dissociation constants for UDP-GlcNAc, 6, 35 and 217 μM, when the concentration of UDP-GlcNAc varies from 0.05 μM to 4.8 mM. OGT enzymatic activity was also shown to be enhanced in response to elevated levels of UDP-GlcNAc [39]. The truncated form of OGT lacking TPRs 1–6 retains comparable enzymatic activity toward casein kinase II (CKII) peptide glycosylation, but it has only two dissociation constants, 6 and 60 μM. These results suggest that OGT activity is regulated by not only the concentration of UDP-GlcNAc but also the subunit structure and composition [39].

In addition to facilitating the formation of OGT trimer, TPRs also help OGT to recognize protein substrates. For example, GABA_A_ receptor-associated protein (GRIF-1) and trafficking Kinesin Protein 1 (TRAK1) were identified as the OGT interacting proteins from yeast two-hybrid screening and found to bind the TPR domain of OGT [41]. OGT has more than 4000 protein substrates. The crystal structure of human OGT may help explain the role of TPRs in binding substrates. A ladder of asparagine on the inner surface of the OGT TPR superhelix accounts for the interactions with various substrates [40]. The mutation of asparagine into alanine on the TPR asparagine arrays results in decreasing *O*-GlcNAcylation [42–44]. These studies provide the evidence that TPR domains of OGT serve as the docking sites for protein substrates. In addition to substrate recognition by TPRs, interaction between the OGT catalytic cleft and the acceptor peptide is also pivotal for OGT to select its substrates [45, 46]. Crystal structure analyses of human OGT combined with various peptides demonstrated that each substrate conjugates with OGT in a similar conformation between the − 3 to + 2 subsites. The size of amino acid and conformational restriction can lead to spatial constraints for the placement of peptides into the catalytic cleft of OGT. Therefore, the peptide sequence specificity, determined from − 3 to + 2 subsites, accounts for stable spatial conformation to interact with the catalytic cleft of OGT [46]. In summary, structural analyses have provided a mechanistic insight into how OGT uses each domain to accommodate and select various protein substrates.

OGA is mainly enriched in the cytosol, unlike OGT, which can accumulate in the nucleus and cytosol [47, 48]. The OGA transcript encodes a protein with three distinct regions consisting of an N-terminal catalytic domain, a stalk domain and a C-terminal pseudo-histone acetyltransferase (HAT) domain [49, 50]. The molecular weight of full-length OGA is about 130 kDa. OGA mRNA also undergoes alternatively splicing in the HAT domain, which results in the encoding of a shorter form of OGA (sOGA). sOGA is approximately 100 kDa and targets to the nascent lipid droplet [51]. Like OGT, OGA is capable of binding to several different protein substrates to exert its functions. OGA is highly conserved among species [47]. Studies of the crystal structure of OGA combined with glycoprotein substrates have suggested that OGA possesses a highly conserved putative groove for substrate docking [49]. The crystal structure of human OGA also provides the first evidence of how mammalian OGA interacts with different substrates [50, 52–54]. Human OGA tends to form a homodimer, and uses a unique substrate-recognition mode, in which the stalk domain of OGA combines with the catalytic domain from the other OGA monomer to form a cleft for substrate binding [50, 52–54]. Furthermore, the inner surface of the substrate binding cleft is mainly composed of hydrophobic residues, which are conserved in most eukaryotes. These hydrophobic interactions are important for spatial constraints and protein binding; consistently, mutation of these residues causes reduced binding of human OGA to the substrates [50]. Structural analyses of the interaction between human OGA and different glycopeptides, including those from α-crystallin, TAB1, ELK1 and lamin B1, showed that GlcNAc moiety is anchored to the conserved residues of the OGA catalytic pocket [52]. This binding mode makes OGA able to select and stabilize *O*-GlcNAcylated peptides in the cleft. Surprisingly, these glycopeptides bind with OGA in a bidirectional manner with identical binding conformations, in spite of their different glycosylation residues and flanking sequences [52]. Together, these studies illustrate that OGA uses a new substrate-binding method to achieve diverse protein recognition.

### Main text

#### *O*-GlcNAcylation regulates the functions of proteins

*O*-GlcNAcylation modifies the S/T residues, which overlap the protein phosphorylation sites, showing that *O*-GlcNAcylation can be a regulatory mode for phosphorylation. Indeed, the “Ying-Yang model” was proposed to illustrate the mode of interplay of these two post-translational modifications, which represents how *O*-GlcNAcylation competes with phosphorylation to occupy the same site or adjacent positions via steric hindrance [55]. For instance, through a kinetic-based high resolution mass spectrometry assay, a frequently occurring phosphorylation/*O*-GlcNAcylation interplay motif, (pS/pT)P(V/A/T)(gS/gT), was identified [56]. Further, phosphorylation on the − 3 subsite hampers *O*-GlcNAcylation on the 0 subsite [56]. Nevertheless, global analyses of the reciprocal effects between *O*-GlcNAcylation and phosphorylation by high throughput mass spectrometry revealed the complexity of the crosstalk [57, 58]. For example, among 711 phosphopeptides identified from NIH-3T3 cells, 208 phosphorylation sites and 148 phosphorylation sites were reduced and increased, respectively, after elevation of *O*-GlcNAcylation levels by treating cells with OGA inhibitors [57]. Further, 10 and 19 proteins showed increased and reduced *O*-GlcNAcylation levels, respectively, after inhibition of glycogen synthase kinase-3 (GSK3) by lithium treatment [58]. These results show that *O*-GlcNAcylation and phosphorylation do not always occur reciprocally. Further, the interplay between *O*-GlcNAcylation and phosphorylation can be at the enzyme level. OGT or OGA modified by phosphorylation on their catalytic subunits or their regulatory subunits affects their activities. For example, tyrosine phosphorylation of OGT resulting from insulin signaling increases the activity of OGT [59]. *O*-GlcNAcylation may regulate kinases or phosphatases, causing indirect changes on the phosphorylation [55, 57]. Many kinases were identified as *O*-GlcNAcylated proteins. The in vitro OGT assay using human kinase array has identified that 39% of the kinases on the array were the OGT substrates [60]. Recently, results from kinase array indicated that more than 80% of human kinases were the substrates of OGT, and over 100 kinases could be *O*-GlcNAcylated in living cells [61, 62]. For example, the increased IKKβ activation caused by *O*-GlcNAcylation on S733 can enhance glycolysis, which further contributes to more elevated *O*-GlcNAcylation and a positive feedback loop between IKKβ and *O*-GlcNAcylation [63]. Interestingly, previous studies demonstrated that the ATPase subunits from the 19S regulatory complex of the proteasome were *O*-GlcNAcylated [64], resulting in the inhibition of ATPase activity and of proteasome mediated proteolysis of Sp1 [65]. Several *O*-GlcNAcylation sites were also identified in the 20S core complex, which implicates that *O*-GlcNAcylation may directly affect proteasome catalytic activity [66]. Therefore, *O*-GlcNAcylation affects many biochemical properties of protein substrates, including protein phosphorylation and stability. Furthermore, *O*-GlcNAcylation can also influence other types of post-translational modification, such as acetylation, which further extends the functions of *O*-GlcNAcylation. For example, increased intracellular *O*-GlcNAcylation results in increased acetylation on Lys (K)382 of p53, which further enhances nuclear translocation of p53 and upregulates the expression of p53 target genes [67]. The p300-mediated acetylation on K310 of p65 of the NF-κB subunit was enhanced by *O*-GlcNAcylation of p65 on T305 and S319, which is important for NF-κB regulated gene expression and cell viability [68, 69].

**Fig. 2:**
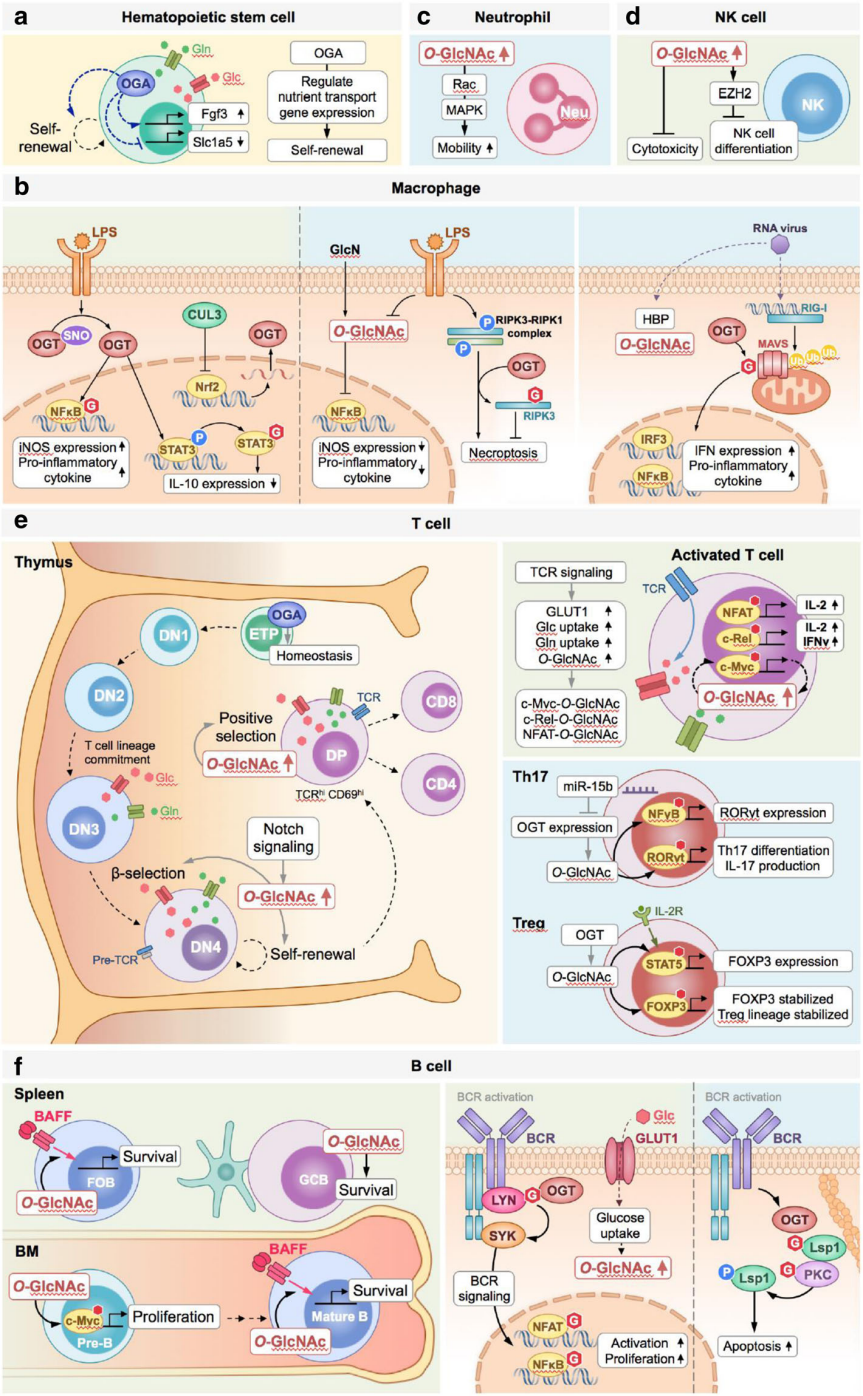
*O-*GlcNAcylation orchestrates immunity. **a** HSCs are able to self-renew and differentiate into all blood cell lineages. OGA controls the homeostasis of *O*-GlcNAcylation, which affects the gene transcription, such as fibroblast growth factor 3 (*Fgf3*) and solute carrier family 1, member 5 (*Slc1a5*), to regulate self-renewal and nutrient transport of HSCs. **b** In macrophages, OGT is de-nitrosylated after LPS treatment, which results in *O-*GlcNAcylation and activation of NF-κB. *O-*GlcNAcylation of STAT3 inhibits its phosphorylation, leading to decreased IL-10 production and increased pro-inflammatory cytokine production (left panel). In certain scenarios, *O-*GlcNAcylation also has an anti-inflammatory function in macrophages. GlcN-induced hyper-*O-*GlcNAcylation inhibits NF-κB-mediated iNOS expression. Moreover, *O-*GlcNAcylation of RIPK3 inhibits RIPK3-RIPK1 complex formation and thus reduces necroptosis-induced inflammation. The antiviral response of macrophages is also regulated by *O-*GlcNAcylation (middle panel). Upon RNA virus infection, MAVS is modified by OGT, which is essential for K63-linked ubiquitination-mediated MAVS activation. This biochemical reaction enhances downstream IFN production via RIG-I signaling (right panel). **c***O-*GlcNAcylation is rapidly increased after neutrophils are activated, which promotes the chemotaxis and cellular mobility of neutrophils. **d***O-*GlcNAcylation may inhibit NK differentiation by increasing the stability of EZH2. In addition, *O-*GlcNAcylation seems to reduce the cytotoxic activity of NK cells. **e** During T cell development in thymus, *O*-GlcNAcylation is required for homeostasis of ETPs. Notch signaling promotes the uptake of glucose (Glc) and glutamine (Gln), which leads to protein *O-*GlcNAcylation, enhanced β selection, and rapid self-renewal of DN4. After TCR rearrangement, *O-*GlcNAcylation promotes positive selection and mature single positive T cell development (left panel). Protein *O-*GlcNAcylation is increased when T cells are activated. *O-*GlcNAcylation is required for activation of many transcription factors, such as NFAT, c-Rel and c-Myc in activated T cells (upper right panel). Notably, *O-*GlcNAcylation also increases the expression of RORγt and FOXP3 in Th17 and Treg cells (lower right panel). **f** In B cell lineages, *O-*GlcNAcylation is upregulated in pre-B cells, thereby promoting the proliferation of pre-B cells through elevating c-Myc expression. Moreover, *O-*GlcNAcylation regulates BAFF signaling to maintain homeostasis of mature B cells in spleen and bone marrow (left panel). When B cells are activated through BCR signaling, Lyn is modified by OGT, which recruits SYK and activates BCR downstream signaling. NFAT and NFκB are activated by *O-*GlcNAcylation in activated B cells, which mediates B cell proliferation. *O-*GlcNAcylation is also involved in BCR crosslinking-induced apoptosis. *O-*GlcNAc modification of LSP1 recruits PKCβ1, which phosphorylates LSP1 and contributes to B cell apoptosis (right panel)

As mentioned above, *O*-GlcNAcylation has been extensively studied in cancer and neurodegenerative diseases [70, 71]. *O*-GlcNAcylation is highly elevated in the majority of cancers as *O*-GlcNAcylation integrates the nutrient flux with the metabolic pathways, which is critical for the proliferation and growth of tumor cells. *O*-GlcNAcylation regulates many proteins involved in cancer initiation and proliferation. For example, *O*-GlcNAcylation increases the stability of p53 and estrogen receptor-β (ER-β) [72, 73]. *O*-GlcNAcylation of c-Myc at T58 residue may promote nuclear localization of c-Myc [74]. *O*-GlcNAcylation of transcription factor YY1 impeded its association with RB, promoted the binding of YY1 with DNA, and may affect cell cycle transitions [75]. In the mammalian brain, *O*-GlcNAcylation of Tau decreases the phosphorylation and cytotoxicity of Tau [76]. The amyloid precursor protein (APP) in Alzheimer’s disease (AD) pathology is modified by *O*-GlcNAcylation [77]. Enhanced *O*-GlcNAcylation increased the processing of the neuroprotective form of APP [78]. This review focuses on the recent advances in the biological and molecular impact of *O*-GlcNAcylation in the immune system (Fig. 2). The role of *O*-GlcNAcylation in each immune cell lineage is discussed separately below.

#### Role of *O*-GlcNAcylation in hematopoietic stem cells

Hematopoietic stem cells (HSCs) are the multipotent stem cells, which reside in bone marrow, and are able to self-renew and differentiate into all blood cell lineages. Conditional knockout of *Oga* in HSCs resulted in the reduced self-renewal of HSCs and decreased bone marrow progenitor populations [79]. RNA-Seq analysis of *Oga* depleted hematopoietic progenitor cells showed that the expression of nutrient uptake and signaling genes are dysregulated [79]. As high rate of glycolysis was used by HSCs to generate energy and maintain the stemness [80], these results suggest that homeostasis of *O*-GlcNAcylation is required for the maintenance of HSCs. These findings also suggest that dysregulation of *O*-GlcNAcylation may impair the development of all types of immune cells.

#### Role of *O*-GlcNAcylation in macrophages

Macrophages are myeloid immune cells and are widely distributed in tissues. In response to foreign pathogens and danger signals within the tissue microenvironment, macrophages initiate innate immune responses and inflammation [81, 82]. Several studies demonstrated that *O-*GlcNAcylation promotes inflammatory responses in macrophages [83–86]. NF-κB, a critical regulator of inflammatory responses in macrophages, is reported to be modified by *O*-GlcNAcylation. Allison et al. showed that OGT could co-localize to the promoter sites regulated by NF-κB and modified T305 of RelA, which promoted acetylation on K310 of RelA, and enhanced NF-κB transcriptional activity after tumor necrosis factor (TNF) stimulation [68]. A previous study showed that c-Rel, a NF-κB subunit, was modified by *O*-GlcNAcylation on S350, which is required for its DNA binding and transactivation activity [87]. Microglia is the brain resident macrophage [88]. In BV2 microglia cells, c-Rel interacts with OGT upon lipopolysaccharide (LPS) treatment, which promotes the *O*-GlcNAcylation of c-Rel and formation of the c-Rel-p50/p105 heterodimeric complex [89]. Another report demonstrated that OGT is modified by S-nitrosylation, which inhibits the catalytic activity of OGT in resting RAW 264.7 murine macrophage cells (Fig. 2a, left panel) [86]. When stimulated with LPS, OGT is de-nitrosylated, which enhances the *O*-GlcNAcylation of p65 [86]. Attenuation of *O*-GlcNAcylation negatively regulates p65 nuclear translocation, resulting in decreased production of NO and interleukin (IL)-1β [86]. STAT3 also serves as a critical transcription factor in promoting inflammation and tissue repair [90, 91]. STAT3 can also induce IL-10 production in macrophages to suppress inflammation [92, 93]. It was previously shown that *O*-GlcNAcylation of STAT3 at T717 negatively regulates STAT3 phosphorylation and reduces IL-10 production [84]. Further, in bone-marrow-derived macrophages (BMDMs), the expression of OGT can be transcriptionally downregulated by myeloid-derived cullin 3 (CUL3), an E3 ubiquitin ligase belonging to the Cullin-RING ligase superfamily through nuclear factor E2–related factor-2 (Nrf2) [84, 94–96]. Therefore, CUL3 counteracts STAT3 *O*-GlcNAcylation, elevates STAT3 phosphorylation and inhibits inflammation (Fig. 2a, left panel) [84].

In contrast, several studies indicated that hyper-*O*-GlcNAcylation correlated with anti-inflammation in ischemia and sepsis [97, 98]. Upon glucosamine (GlcN) treatment, which bypasses the GFAT rate-limiting step and induces hyper-*O*-GlcNAcylation [21], the transcription activity of c-Rel is inhibited, thereby reducing the NF-κB-mediated expression of inducible nitric oxide synthase (iNOS) in LPS stimulated BV2 microglia cells (Fig. 2a, middle panel) [89, 98]. Moreover, it has been recently shown that *O*-GlcNAcylation is capable of interacting with receptor-interacting serine/threonine-protein kinase 3 (RIPK3), a member of the necrosome complex [99], to inhibit necroptosis of macrophages and reduce necroptosis-induced cytokine production and inflammation (Fig. 2a, middle panel) [100]. *O*-GlcNAcylation of RIPK3 at T467 suppressed RIPK3-RIPK1 and RIPK3-RIPK3 complex formation, subsequently inhibiting necroptosis of macrophages [100]. *Ogt*^*f/f*^* Lyz2*-cre conditional knockout mice, which carry deleted *Ogt i*n macrophages showed significantly increased RIPK3 activation, elevated inflammatory cytokine production, and more severe mortality in experimental sepsis, as compared with control mice [100]. Furthermore, *O*-GlcNAcylation may direct the polarization of M2 macrophages, which contribute to inflammation resolution and tissue repair [97, 101, 102]. A recent study showed that GlcN treatment reduced M1 specific gene expression in macrophages in an LPS-induced septic lung injury animal model [97]. Accordingly, administration of Thiamet-G, a specific OGA inhibitor [103], increases the expression of M2 markers on microglia and suppresses NF-κB p65 signaling, which leads to the reduction of iNOS and cyclooxygenase-2 (COX-2) expression after middle cerebral artery occlusion [102]. Therefore, changes in *O*-GlcNAcylation levels may alter the differentiation of M2 vs. M1 macrophages in tissues.

Antiviral immunity of macrophages was also shown to be regulated by *O*-GlcNAcylation [83, 85]. Administration of GlcN protects mice from RNA virus infections, such as influenza virus, vesicular stomatitis virus and coxsackievirus A6 [85]. Depletion of *Ogt* in macrophages by *Lyz2*-cre abolished this protective effect, indicating that *O*-GlcNAcylation is required for anti-RNA virus infection [83, 85]. Both retinoic acid inducible gene I (RIG-I) and melanoma differentiation associated gene 5 (MDA5) can respond to RNA virus infection [104]. After RIG-I and MDA5 activation, tripartite motif-containing protein 31 (TRIM31) induces the activation of mitochondrial antiviral signaling protein (MAVS), a crucial adaptor protein for antiviral responses [105, 106], via K63-linked ubiquitination [104, 107]. MAVS also promotes activation of interferon regulatory factor 3 (IRF3) and NF-κB, leading to the production of type I interferon (IFN) and cytokines to against virus infection [104, 106]. MAVS is modified by *O*-GlcNAcylation, which promotes RIG-I and MDA5 mediated IRF3 activation as well as IFN production in RNA virus infections (Fig. 2a, right panel) [85]. Song, et al. showed that multiple *O*-GlcNAcylation at 322–347 amino acid regions of MAVS is essential for MAVS activation and IFN signaling [85]. S366 of MAVS is *O*-GlcNAcylated after a RNA virus infection, which functions upstream of the TRIM31 mediated K63-linked ubiquitination of MAVS, and the activation of IRF3 and NF-κB in BMDMs [83]. In addition, MAVS conditional knockout mice also showed a diminished protective effect of GlcN [85]. To summarize, *O*-GlcNAcylation can promote antiviral responses via regulating MAVS activity in macrophages.

As mentioned above, *O*-GlcNAcylation appears to have opposite functions in inflammatory responses in macrophages. Although it requires further study, this discrepant effect of *O*-GlcNAcylation in macrophages could be related to M1 vs M2 polarization, or tissue residency. Nevertheless, results from the study in *Caenorhabditis elegans* may give further explanation. *Oga-1* and *ogt-1* knockout nematodes display similar, but not contrary, phenotype in insulin-like signaling [108, 109], suggesting that OGT and OGA seem to coordinately regulate the level of intracellular *O*-GlcNAcylation. Therefore, an optimal zone of *O*-GlcNAc level is critical for the maintenance of normal cellular function [110]. In addition, the binding of c-Rel to *iNOS* promoter is changed depending on the concentrations of glucose in the culture [111], implying that optimal range of *O*-GlcNAcylation is important for shaping gene expression in inflammatory responses. Thus, another possibility is that both hyper- and hypo-*O*-GlcNAcylation may cause immune deregulation.

#### Role of *O*-GlcNAcylation in neutrophils

Neutrophils are polymorphonuclear leukocytes, which rapidly infiltrate wounds and lesion sites during infection or tissue damage. Neutrophils not only phagocytose pathogens and produce pro-inflammatory cytokines, ROS and granular proteins, but also release neutrophil extracellular traps (NET) to minimize the damage caused by pathogens [112]. Previous studies demonstrated that *O-*GlcNAcylated proteins in neutrophils are rapidly increased within 2 min following treatment with *N*-Formylmethionine-leucyl-phenylalanine (fMLF), a polymorphonuclear and mononuclear phagocyte activator [113–115], suggesting that *O-*GlcNAcylation may be crucial for the function of neutrophils. Increased *O-*GlcNAcylation promotes chemotaxis and cellular mobility of neutrophils [114, 116]. Meanwhile, administration of PUGNAc, an OGA inhibitor [4, 117], or GlcN upregulates the activity of Rac, an important small GTPase for regulating neutrophil mobilization [118], and activates downstream p38 and p44/42 MAPK signaling [116]. In summary, these results indicate that *O-*GlcNAcylation promotes neutrophil mobilization via increasing Rac activation (Fig. 2b).

#### Role of *O*-GlcNAcylation in NK cells

Natural killer (NK) cells are innate lymphoid cells. They are cytotoxic effector cells often involved in tumor surveillance and infection clearance [119]. Enhancer of zeste homolog 2 (EZH2), which is a histone methyltransferase in the polycomb repressive complex 2 (PRC2), limits the differentiation and survival of NK cells [120, 121]. It has been shown that *O*-GlcNAcylation of EZH2 at S75 promotes the stability of EZH2 [122]. Moreover, previous studies demonstrated that *O*-GlcNAcylation may regulate cytotoxicity of NK cells [123, 124]. Protein *O*-GlcNAcylation is down-regulated in NK cells during cytotoxicity execution; while, inhibition of cytotoxic activity by treating NK cells with recombinant soluble HLA-G1, a nonclassical MHC I molecule [125], enhanced *O*-GlcNAcylation levels in NK cells [123]. In addition, treatment with GlcN reduced the cytotoxic activity of NK cells, which is accompanied by the altered localization of cathepsins C and E, and polarization of lytic granules in NK cells [124]. Interestingly, GlcN treatment in NK cells reduced phosphorylation of FOXO1 (a negative regulator of NK cell function [126]), and paxillin, but elevated *O*-GlcNAcylation of FOXO1 and paxillin. Although the detailed mechanisms of how *O*-GlcNAcylation affects the function of FOXO1, paxillin and other proteins in NK cells remains to be studied, these results suggest that *O*-GlcNAcylation negatively regulates the cytotoxic effects of NK cells (Fig. 2c).

#### Role of *O*-GlcNAcylation in T cells

Rapid cycling of *O*-GlcNAc modification is detected in activated lymphocytes [127]. *O*-GlcNAcylation has been reported to play an important role in T cell lineages. T cell progenitors travel from bone marrow to the thymus and differentiate to early thymic progenitors (ETPs). In the thymus, ETPs commit to the T cell lineage and become double negative (DN) cells, which further complete T cell development through various checkpoints at different stages. A recent study shows that ETPs is decreased in *Oga*^*f/f*^*Vav*-cre mice, in which *Oga* is deleted in HSCs. These results indicate that *O*-GlcNAc homeostasis is essential for very early stages of T cell development [79]. Successful rearrangement of the T cell receptor (TCR) β chain enables the presence of a pre-TCR complex on the cell surface, which mediates β-selection and promotes the development of DN3 to DN4 [128–131]. As a result, DN4 cells show potential for self-renewal and finally become double positive (DP) thymocytes. Swamy et al. showed that Notch signaling, which is required for the β-selection checkpoint in the DN3 to DN4 transition [132, 133], significantly increases the import of glucose and glutamine when DN3 thymocytes differentiate into DN4 thymocytes (Fig. 2d, left panel) [24]. After becoming DP thymocytes, the uptake of glucose and glutamine is downregulated [24]. In parallel with nutrient uptake, protein *O*-GlcNAcylation in developing thymocytes was increased at the DN4 stage [24]. It is noted that Notch signaling-mediated upregulation of *O*-GlcNAcylation is important for β-selection checkpoints. Consistently, depletion of *Ogt* in developing thymocytes resulted in the reduction of DP thymocytes, revealing the importance of *O*-GlcNAcylation in developing thymocytes during the transition into the DP stage [24]. In addition, in vitro results indicated that *Ogt*-depleted DN cells have normal differentiation and survival, but they failed to proliferate in response to Notch signaling [24]. These results suggest that *O*-GlcNAcylation is essential for self-renewal of DN4. DN4 cells initiate TCR α chain rearrangement to produce a TCR complex [134], which allows the proceeding of positive selection and negative selection to become single positive (SP) CD4 or CD8 T cells [134]. Protein *O*-GlcNAcylation is increased during positive selection (Fig. 2d, left panel) [24]. Moreover, both CD4 and CD8 SP cells dramatically decrease in T cell-specific *Ogt* knockout mice [24]. Thus, *O*-GlcNAcylation is also required for positive selection of T cells.

*O*-GlcNAcylation also plays a crucial role in mature T cells. Activated T cells, triggered by treatment with anti-CD3/anti-CD28 antibodies, increase the expression of glucose transporter 1 (GLUT1), a membrane transporter that mediates glucose uptake and further causes a dramatic increase in glucose uptake [135]. Hence, activated T cells rapidly increase uptake of glucose and show elevated levels of protein *O*-GlcNAcylation (Fig. 2d, upper right panel) [24]. Numerous studies have indicated that *O*-GlcNAc modification of many transcription factors participates in the regulation of T cell activation. For instance, nuclear factor of activated T cells (NFAT), a key regulator for IL-2 and cytokine production by T cells [136], interacts with OGT and is *O*-GlcNAcylated [137]. Downregulation of OGT results in impaired NFAT function and inhibits IL-2 production [137]. In contrast, *O*-GlcNAcylation of c-Rel, a NF-κB subunit, at S350 is required for its DNA binding and transcriptional activation [87]. Mutation at S350 of c-Rel reduces the expression of NF-κB downstream targets, including cytokine (IL-2 and IFNγ), in response to TCR activation [87]. Further, c-Myc, a critical metabolic regulator [138], is modified by *O-*GlcNAc [24, 139]. OGT promotes c-Myc expression in T cells during expansion. Depletion of c-Myc in T cells impaired the expression of glucose and glutamine transporters upon T cell activation [138]. Moreover, knockout of *c-Myc* in T cells abolishes the induction of TCR-mediated *O*-GlcNAcylation [24]. These data suggest that c-Myc mediates a positive feedback loop between TCR-mediated T cell activation and protein *O*-GlcNAcylation.

After TCR activation, depending on the environmental cues, naïve CD4 T cells can differentiate to various types of effector cells, such as T helper (Th)1, Th2, Th17 and regulatory T (Treg) cells. Recent studies indicated that *O*-GlcNAcylation is implicated in both differentiation and homeostasis of Th17 and Treg cells (Fig. 2d, lower right panel) [140–142]. Administration of Thiamet-G increased the binding of RORγt, a pivotal transcription factor for commitment of the Th17 lineage [143], to *Il-17* promoter and therefore promotes IL-17 production and Th17 differentiation. Subsequently, pro-inflammatory responses were enhanced by Th17 cells [142]. OGT is a downstream target of microRNA(miRNA)-15b [140]. miRNA-15b inhibits Th17 differentiation, which may result from reducing the expression of RORγt through blocking *O*-GlcNAcylation of NF-κB [140]. FOXP3 is a lineage-determining transcription factor for Treg cells [144, 145]. It has been reported that FOXP3 is stabilized by *O*-GlcNAcylation, and that *O*-GlcNAcylation is required for IL-2/STAT5 signaling-mediated FOXP3 expression. Depletion of *Ogt* in Treg cells in mice dramatically reduced Treg lineage stability, which resulted in a severe autoimmune phenotype [141]. Thus, *O*-GlcNAcylation is required for the maintenance of lineage stability and regulatory function of Treg cells. IL-2 signaling-mediated STAT5 activation is also important for Th2 differentiation. STAT5, which binds to *Il-4* gene, promotes optimal production of IL-4 and Th2 differentiation [146]. *O*-GlcNAcylation may thus likely regulate Th2 differentiation through IL-2/STAT5 signaling, although it requires further validation. In addition, IFN-γ-induced STAT1 activation is required for the induced expression of Th1 lineage-determining transcription factor, T-bet [147]. Previous studies indicated that *O*-GlcNAcylated STAT1 enhanced the stability of phosphorylated STAT1 and its downstream signaling transduction [148, 149]. Whether *O*-GlcNAcylation of STAT1 participates in the regulation of Th1 differentiation needs to be further determined. The functional role of *O*-GlcNAcylation in CD8 T cells awaits further characterization. *O*-GlcNAc-enriched proteome profiling of murine effector and memory-like CD8^+^ T cells has been reported [150], which showed that protein *O*-GlcNAcylation in effector CD8^+^ T cells is involved in transcription and translation essential for the regulation of cell proliferation, while protein *O*-GlcNAcylation in memory-like CD8^+^ T cells is involved in the mRNA processing. Taken together, these combined findings indicate that *O*-GlcNAcylation controls the development, activation and differentiation of a variety of T cell subsets at various stages.

#### Role of *O*-GlcNAcylation in B cells

B cells contribute to adaptive immune responses via producing antibodies and promoting T cell activation via antigen presentation. Progenitor B cells undergo pro-B, early pre-B and late pre-B stages to become immature B cells in bone marrow. A recent study indicated that *O-*GlcNAcylation is increased in large pre-B cells and required for the proliferation of pre-B cells [151]. Inhibition of *O*-GlcNAcylation by administration of OGT inhibitors downregulates the expression of c-Myc, cyclin A and cyclin E in a PD36 pre-B cell line. These results suggest that *O*-GlcNAcylation induces pre-B cells proliferation by increasing the expression of c-Myc and c-Myc downstream genes, such as cyclin A and cyclin E [151].

Immature B cells then migrate to secondary lymphoid organs, including the spleen, in which they differentiate into mature B cells, including marginal zone B (MZB) cells and follicular B (FOB) cells. Deletion of *Ogt* in B cells by using a CD19 promoter-driven cre mouse line showed that *O-*GlcNAcylation is required for the homeostasis of mature B cells, particularly FOB cells (Fig. 2e, left panel) [152]. Specific depletion of OGT in B cell lineages resulted in downregulation of B cell-activating factor (BAFF) signaling in mature B cells, which lead to increased apoptosis of mature B cells in bone marrow and spleen. However, the number of immature B cells and MZB are not significantly influenced by the depletion of *Ogt* [152]. Notably, upon B cell receptor (BCR) engagement-mediated B cell activation, metabolic reprograming induces the expression of GLUT1 (Fig. 2e, right panel) [153]. Consistently, the *O*-GlcNAcylation level in activated B cells, caused by ligation of BCR, is elevated, which is required for efficient B cell activation [137, 152, 154]. NFATc1 and NF-κB are modified by *O*-GlcNAc, which further strengthens the extent of B cell activation [137]. In addition, *O*-GlcNAcylation of Lyn on S19 is important for Lyn activation and Syk recruitment. Results from studies of the role of *O*-GlcNAcylation in BCR signaling cascades support the role of *O*-GlcNAcylation in B cell survival (Fig. 2e, right panel) [152]. In the context of extensive BCR crosslinking and absence of CD40L-mediated survival signals provided by T cells, activated B cells undergo apoptosis. *O*-GlcNAcylation is also involved in the regulation of BCR crosslinking-induced apoptosis. Lymphocyte specific gene 1 (LSP1) is known to mediate anti-IgM-induced B cell apoptosis [155]. The dynamic interplay between *O*-GlcNAcylation and phosphorylation of LSP1 following BCR crosslinking determines the apoptosis of activated B cells. *O*-GlcNAcylation of Lsp1 on S209 is critical for the recruitment of PKC-β1, which contributes to phosphorylation of Lsp1 on S243 [154]. As a result, *O-*GlcNAcylation promotes signaling cascades and apoptosis of activated B cells (Fig. 2e, right panel) [154].

In the help with T follicular helper (Tfh) cells, activated B cells differentiate to germinal center B (GCB) cells, which undergo somatic hypermutation and affinity maturation within GCs, followed by further differentiation of GCB cells into antibody secreting plasmablasts/plasma cells or memory B cells. *O*-GlcNAcylation also plays a crucial role in the differentiation of GCB cells. Studies from the mouse lines carrying a deletion of *Ogt* in the GC stage revealed that the generation of GCB cells and plasma cells requires *O*-GlcNAcylation [152]. However, the detailed molecular mechanisms contributing to the reduced generation of plasma cells after deletion of *Ogt* in GCB cells requires further study. In conclusion, in B cell lineages, *O*-GlcNAcylation is pivotal for the regulation of homeostasis and activation of mature B cells, as well as for mounting efficient GC and antibody responses.

## Conclusions

*O*-GlcNAcylation, mediated by the dynamic coordination of the actions of OGT and OGA, contributes to the regulation of the development, homeostasis, and functions of immune cells. Dysregulated *O*-GlcNAc cycling, as shown by lineage specific knockout of *Ogt* or *Oga* in mice has demonstrated the significance of *O*-GlcNAcylation in a variety of immune cells, including HSCs, T cells, B cells, NK cells, macrophages, and neutrophils. However, applying genetic deletion of *Ogt* or *Oga*, or inhibitor treatment, to study the role of *O*-GlcNAcylation in a biological system still raises the challenge of understanding of the impact of site-specific *O*-GlcNAcylation as site-directed mutagenesis of protein *O*-GlcNAc sites from S/T into alanine to abolish the *O*-GlcNAcylation event may as well dampen phosphorylation event. Intriguingly, a recent report showed that the engineered cysteine-S-GlcNAc is a hydrolytically stable and accurate structural mimic of serine-O-GlcNAc. Therefore, the potential effect of site-specific *O*-GlcNAcylation on a selected protein can be assessed through replacing serine with cysteine on the identified *O*-GlcNAc sites [156]. Moreover, many studies used GlcN to induce hyper-*O*-GlcNAcylation. However, GlcN treatment affects not only *O*-GlcNAcylation but also *N*-linked glycosylation [157]. Therefore, the consequence of altered *N*-linked glycosylation resulting from GlcN treatment should also be taken into consideration.

In terms of autoimmune or inflammatory diseases, increased expression of X-linked genes, including *OGT*, is linked with lupus in women [158]. Lack of *Ogt* in an animal model of autoimmune hepatitis in rats exacerbated liver injury due to impaired Treg differentiation [159]. Therefore, modulation of the levels of *O*-GlcNAcylation may likely control the outcome of diseases, highlighting the alternation of *O*-GlcNAcylation levels as a potential treatment strategy. The development of potent and selective OGT or OGA inhibitors may thus possess potential for the treatment of diseases that show abnormal *O*-GlcNAcylation. Indeed, several OGT or OGA inhibitors have been developed [103, 160–162]. OGA inhibitors have recently entered early clinical trials for the treatment of Progressive Supranuclear Palsy [163] as *O*-GlcNAcylation of Tau blocks the pathological effects of phosphorylation and aggregation of Tau [76]. It remains to be evaluated if modulation of the functions of OGT or OGA can be a good remedy for immune system-related diseases. Nevertheless, the significant roles of *O*-GlcNAcylation in various lineages of immune cells in the physiological state may shed light on the development of new strategies to boost or rejuvenate immune responses against diseases, such as infection or cancer.

## Data Availability

Not applicable.

## Abbreviations

Acetyl-CoA: Acetyl-coenzyme A
AD: Alzheimer’s disease
APP: Amyloid precursor protein
BAFF: B cell-activating factor
BCR: B cell receptor
BMDMs: Bone marrow derived macrophages
CKII: Casein kinase II
COX-2: Cyclooxygenase-2
CUL3: Cullin 3
DN: Double negative
DP: Double positive
ETP: Early thymic progenitor
ER: Endoplasmic reticulum
ER-β: Estrogen receptor-β
EZH2: Enhancer of zeste homolog 2
F-6P: Fructose-6-phosphate
Fgf3: fibroblast growth factor 3
fMLF: *N*-Formylmethionine-leucyl-phenylalanine
FOB: Follicular B
G-6P: Glucose-6-phosphate
GCB: Germinal center B
GFAT: Glutamine:fructose-6-phosphate amidotransferase
GlcN: Glucosamine
GlcN-6P: Glucosamine-6-phosphate
GlcNAc: *N*-acetylglucosamine
GlcNAc kinase, NAGK: *N*-acetylglucosamine kinase
GlcNAc-1P: *N*-acetylglucosamine-1-phosphate
GlcNAc-6P: *N*-acetylglucosamine-6-phosphate
Glc: Glucose
GLUT1: Glucose transporter 1
Gln: Glutamine
GNPNAT, EMeg32: Glucosamine-phosphate *N*-acetyltransferase
GPI: Phosphoglucose isomerase
GRIF-1: GABA_A_ receptor-associated protein
GSK-3: Glycogen synthase kinase-3
HAT: Histone acetyltransferase
HBP: Hexosamine biosynthetic pathway
HK: Hexokinase
HSCs: Hematopoietic stem cells
IFN: Interferon
IL: Interleukin
iNOS: Inducible nitric oxide synthase
IRF3: Interferon regulatory factor 3
K: Lysine
LPS: Lipopolysaccharides
LSP1: Lymphocyte specific gene 1
MAVS: Mitochondrial antiviral signaling protein
MDA5: Melanoma differentiation associated gene 5
miRNA: MicroRNA
mOGT: Mitochondrial OGT
MZB: Marginal zone B
ncOGT: Nucleocytoplasmic OGT
NET: Neutrophil extracellular traps
NFAT: Nuclear factor of activated T cells
NK: Nature killer
Nrf2: Nuclear factor E2–related factor-2
OGA: *O*-GlcNAcase
*O*-GlcNAcylation: *O*-linked-N-acetylglucosaminylation
OGT: *O*-GlcNAc transferase
PGM3/AGM1: GlcNAc phosphomutase
PPi: Pyrophosphate
PRC2: Polycomb repressive complex 2
RIG-I: Retinoic acid inducible gene I
RIPK3: Receptor-interacting serine/threonine-protein kinase 3
S: Serine
Slc1a5: Solute carrier family 1, member 5
sOGA: Shorter form of OGA
sOGT: Shortest form of OGT
SP: Single positive
T: Threonine
TCR: T cell receptor
Tfh: T follicular helper
Th: T helper
TNF: Tumor necrosis factor
TPRs: Tetratricopeptide repeats
TRAK1: Trafficking Kinesin Protein 1
Treg: Regulatory T
TRIM31: Tripartite motif-containing protein 31
UAP1/AGX1: UDP-GlcNAc pyrophosphorylase
UDP-GlcNAc: Uridine diphosphate *N*-acetylglucosamine
UTP: Uridine-5′-triphosphate
